# The changing distribution of *Leishmania infantum* Nicolle, 1908 and its Mediterranean sandfly vectors in the last 140 kys

**DOI:** 10.1038/s41598-019-48350-7

**Published:** 2019-08-14

**Authors:** Attila J. Trájer, Viktor Sebestyén

**Affiliations:** 1 University of Pannonia, Department of Limnology, H-8200 Veszprém, Egyetem utca 10, Hungary; 2 University of Pannonia, Institute of Environmental Engineering, H-8200 Veszprém, Egyetem utca 10, Hungary

**Keywords:** Biogeography, Parasitic infection

## Abstract

The understanding of the effects of past climatic changes on the distribution of vector arthropods can strongly support the understanding of the future potential impact of anthropogenic climatic change on the geographical risk of vector-borne diseases. The zoogeographical patterns of the European sandfly vectors may suffer the continuously changing climate of the last 140 kys. The former range of *L. infantum* and six *Phlebotomus* species were modelled for the Last Interglacial, the Last Glacial Maximum and the Mid-Holocene Periods. It was found that the potential distribution of the parasite was much smaller in the Last Glacial Period *L. infantum* mainly could persist in the western shelves of the Mediterranean Sea. West and East Mediterranean sandfly species inhabited partly distinct refugia. The Apennine Peninsula, Sicily and the Iberian refugium formed a habitat chain along with the coastal areas of the West Mediterranean Basin. There was no direct connection between the Eastern and the Western sandfly refugia in the last 140 kys. The modelled distribution of sandfly taxa for the Middle Holocene Period can explain the relict populations of sandfly taxa in such Central European countries. The former genetic studies strongly confirm the existence of the modelled glacial refugees.

## Introduction

The present-day climatic change triggers the northward spread of *Leishmania* vector *Phlebotomus* species causing the expansion of leishmaniasis in Western Eurasia^1,2^. To better understand the recent zoogeographical patterns which provide the basis of the modern change of the ranges, the analyses of the past conditions cannot be neglected. It was found that genetically differentiated populations of sandfly species of an area might possibly hinder the northwards spread of leishmaniasis in the recent times^3^. This points out that the late Pleistocene-Holocene history of the sandfly fauna could impact the future expansion of sandfly-borne diseases creating the basis of the recent expansion. Considering the recent geographical range of *Phlebotomus* species, it can be stated that there are more than 700 species worldwide and about 40 species thought to be the vector of different *Leishmania* protozoans^4^. *Leishmania infantum* Nicolle, 1908 is the causative agent of human and canine visceral leishmaniasis in the Mediterranean area^5^. Dogs are the only confirmed primary reservoir of *L. infantum* infection^6^. The transmission of the parasite among dogs can be maintained by several non-sandfly routes, such as intrauterine and sexual transmission. Humans can be infected by sandfly transmission or blood to blood transfer^6,7^.

The following Mediterranean *Phlebotomus* species are the confirmed vectors of *L. infantum*: *Phlebotomus* (*Larroussius*) *ariasi* Tonnoir, 1921^8,9^, *Phlebotomus* (*Larroussius*) *major* subsp. *neglectus* Tonnoir, 1921^10–13^ (since simply: *Ph. neglectus*), *Phlebotomus* (*Phlebotomus*) *papatasi* Scopoli, 1786^14,15^; *Phlebotomus* (*Larroussius*) *perfiliewi* Parrot, 1930^16,17^, *Phlebotomus* (*Larroussius*) *perniciosus* Newstead, 1911^9,18–20^, *Phlebotomus (Larroussius) tobbi* Adler and Theodor, 1930^11,21–23^. The *L. infantum* vector competence of *Phlebotomus* (*Paraphlebotomus*) *alexandri* Sinton, 1928 is supposed in Iran^24^. The *Leishmania* vector competence *of Phlebotomus (Larroussius) mascittii* Grassi, 1908 is not sufficiently known. *Phlebotomus (Paraphlebotomus) sergenti* Parrot, 1917 is the primarily vector of *Leishmania tropica* Wright, 1903^10,25–32^. *Phlebotomus sergenti* and *Phlebotomus similis (Paraphlebotomus)* Perfiliev, 1963 are the vectors of *Leishmania killicki* Rioux, Lanotte & Pratlong 1986 in the Mediterranean region^28,31^.

To investigate whether the populations of sandfly species and the parasites will spread northwards in Europe due to climate change, the modelling of the past climate change triggered zoogeographical changes is inevitable^3^. It is well-known that while the distribution of the potential vectors covers the European range of *L. infantum*, the parasite itself has a much smaller extension^7^. Based on this fact, both the recent and the future modelled ranges of the parasite is smaller than its vectors^1^. The climatic sensibility of the parasite can be the consequence of that the stage differentiation (the promastigote to amastigote differentiation) of *L. infantum* promastigotes is controlled by temperature-sensible heat shock protein 83 gene-mediated transcription process^33^. This implies indirectly that the former range of the parasite was always smaller than the range of the vector sandfly species. The analyses of the glacial and post-glacial change of the distribution of *Phlebotomus* species will help to predict the capacity of this insects to disperse and originate new foci of cutaneous, mucocutaneous or visceral leishmaniasis in response to the anthropogenic climate change^34^.

*Phlebotomus* species are highly sensitive to climatic conditions which make possible to model their distribution using primarily or solely climatic factors. In general, the distribution of the European *Phlebotomus* species corresponds to the 10 °C annual isotherm and July isotherm of 16–18 °C^7^. However, climate envelope models showed that climatic limiting values somewhat differ by species and climatic variables^1,35,36^. Sandfly species are sensitive to the sudden changes of temperature, preferring the warm but moderate climate areas with low interannual and circadian meteorological fluctuations^7^. Larval development and the activity of imagoes strongly depend on temperature and slows down rapidly when the temperature drops below 20 °C^7^, although some *Phlebotomus* species can remain active down to 13 °C^37,38^. In summer, sandfly individuals rest in cool and wet habitats during daytime avoiding the unfavourable hot and dry atmospheric conditions^39^. *Phlebotomus* species breed on the wet bacterial math of rift surfaces or in the barks of trees and find shelters in rodent burrows and cliff cracks in nature^40^. It can be concluded that the lack of standing waters does not limit the distribution of sandflies and temperature and humidity are the most important factors of the activity, development, and survival of sandfly species^7^.

Several genetic studies were performed directly or indirectly to study the effects of the past, mainly glacial or post-glacial climatic changes of the Mediterranean sandfly fauna^3,41,42^. The present climatic limiting factors of the Mediterranean sandfly species trace back to the late Neogene-Pleistocene climatic and geographical changes in Europe^43^. The alternation of the Pleistocene glacial and interglacial periods caused the repeated retreat from the continental areas and the subsequent radiation from the Mediterranean Basin to the northern parts of Europe of sandflies species. The cycles of this retreats and radiations, *Phlebotomus* species suffered a population bottleneck late in the Pleistocene and then radiated out from the Eastern Mediterranean subregion^44^. The above-mentioned authors had valuable conclusions about the Pleistocene history of the Mediterranean sandfly spices, although the synthesis of the genetic results, the recent distribution of the species, the ancient sea level and climatic changes have not been performed yet. The modelling of the late Pleistocene-Middle Holocene distribution of *L. infantum* and its vector sandfly species and the effect of the glacial sea level on the *Phlebotomus* fauna in the Mediterranean area previously were not studied.

Trájer *et al*.^45^ classified the Mediterranean sandfly fauna according to its distribution in Europe, excluding the distribution of *Ph. alexandri* and *Ph. mascittii*. Based on their classification in species level, three kinds of sandfly fauna exist among *L. infantum* vector sandfly species: a West Mediterranean (including *Ph. ariasi*, *Ph. perniciosus*), an East Mediterranean (including *Ph. neglectus*, *Ph. perfiliewi*, *Ph. tobbi*) and a Trans-Mediterranean (including the sole species *Ph. papatasi*). Hence, we will use this collective fauna groups of the studied sandfly species in the sense of the concept of Trájer *et al*.^45^.

## Aims

It was aimed to model the former distribution area of the six confirmed Mediterranean vector *Phlebotomus* species and *L. infantum* for the Last Interglacial Period (120–140 kys BP, hence: LIP), the Last Glacial Maximum (22 kys BP, hence: LGM), during the Min-Holocene Period (6,000 ys BP, hence: MHP), for the ‘current’ period 1961–1990 and the past diversity of the sandfly fauna in the modelled periods.

## Material and Methods

### The source of the used extrema

The temperature and precipitation extrema of *L. infantum* and the six *Phlebotomus* species in the monthly resolution were based on the results of Trájer *et al*.^1^. The authors run iteratively the modelled distribution of eight *Phlebotomus* species and *L. infantum* and compared them to the observed distributions to investigate the optimal number of percentiles to be left from the climatic values. They calculated cumulative distribution functions for the involved temperature and precipitation climatic parameters for the 12 months. From the studied species, *Leishmania infantum* and its confirmed six Mediterranean sandfly vectors were involved to the present study. The vector sandfly species were as follows: *Ph. ariasi*, *Ph. neglectus*, *Ph. papatasi*, *Ph. perfiliewi*, *Ph. perniciosus* and *Ph. tobbi*. The *Phlebotomine* maps menu in the European Centre for Disease Prevention and Control’s (ECDC) website was the source of the distribution data for 2011 of the European *Phlebotomus* species in the study of Trájer *et al*.^1^. The map shows the distribution of *Phlebotomus* sandfly species in Europe, Northernmost Africa, Asia Minor and the Levant at ‘regional’ administrative level (e.g. NUTS3 regions in the European Union. Trájer *et al*.^1^ gained the distribution data for 2001 of *L. infantum* from the publication of Trotz-Williams and Trees^46^.

### The modelled periods and the used climate models

The potential past distribution of the unified area of the involved six *Phlebotomus* species was modelled for the Last Interglacial Period (LIP), for the LGM, for the Mid-Holocene Period (MHP) and for the ‘current’ (reference) period 1961–1990. In the case of the MHP and LGM, the results of the CCSM4 paleoclimate simulation were used. The downscaled data were retrieved from the WorldClim - Global Climate Data database. The monthly average minimum temperature (°C) and the monthly total precipitation sums (mm) were used. The LGP’s climatic model was gained from the published model of Otto-Bliesner *et al*.^47^.

### Distribution limiting climatic values and climate envelope modelling

We aimed to model the past distribution of the selected six vector sandfly species and *L. infantum* with Climate Envelope Modelling (CEM; (also known as niche-based modelling, correlative modelling)^48^. The basic idea of CEM that distribution is mostly dependent on the climatic factors^49^. In the case of e.g. plants, the application of this method is somewhat questionable^50^, although it is known that such non-climatic (edaphic and other) factors as the dominant soil type or the presence of pests and pollinators also strongly impacted by the climate. For the synanthropic sandfly species, the latter factors do not seem to be important, and temperature and precipitation are relevant drivers for the distribution of sandflies^36^.

CEM method involves drawing an envelope around the domain of climate variables where a species currently occurs and then identifying areas predicted to fall within that domain under modelled climates, scenarios for the past, the present or the future^51,52^. CEM tries to find statistical correlations between the range of species and climate factors^53,54^. Several other ways can be found in the literature to determine the potential distribution of animal or plant species^51^. What is an advantage of the used modelling approach in contrast to the more novel, rather machine-based modelling approaches such as the feed-forward artificial neural network modelling or the support vector machines learning mechanism, that the extrema are easy to extract. The CEM methods rarely exclude those observed areas which do not completely fit into the model (the omission error is low). It is also the advantage of this modelling approach that the gained climatic variables easily can be used for further modelling purposes and less dependent on the specific model environment than on other modelling environments.

### Model identification

Modelling results were displayed using GIS (Quantum GIS 3.4.4^55^ with GrassGis7.4.1 software^56^). The Lambert Azimuthal Equal Area (EPSG:3035) was used as projection system. The steps of modelling are as follows: the suitable climatic parameters were selected from the climate extrema results of Trájer *et al*.^1^ for modelling: the monthly minimum temperature (min(Tmean), °C), the monthly minimum precipitation (min(P), mm) and the monthly maximum precipitation (max(P), mm) of the 12 months (see Supplementary Table 1). This means 36 factors in the model in the case of all species. For the numerical modelling of the potential distribution areas of species, the above-described extrema of climatic factors were used. There is a distribution function within the extrema, which shows the distribution maximum for the given factor, but in the proposed approach the distribution of the internal interval is neglected, the appearance of the species is characterized by true or false (0; 1) values according to the Boolean algebra.

Using climatic factors, deterministic unit step functions can be written in the following form:

$$1({T}_{min})=\{\begin{array}{lll}0 & if & {T}_{min}\le {T}_{limit}\\ 1\, & if & {T}_{min} > {T}_{limit}\end{array}$$

$$1(P)=\{\begin{array}{ccc}0 & if & {P}_{limit.max}\le P\le {P}_{limit.min}\\ 1 & if & {P}_{limit.min} < P < {P}_{limit.max}\end{array}$$

where *T*_*min*_ represents the georeferenced climate model data of the monthly average minimum temperature, the *T*_*limit*_ is the lower limitation factor (extremum) of the given species, *P* is the georeferenced climate model data of the monthly precipitation and *P*_*limit.min-max*_ shows the precipitation extrema of the given species. As can be seen, the limitation factor values are climate model independent.

The temperature and precipitation climate models are available in a monthly resolution. The areas excluded by the limiting factors must be summed, and the intersection of the potential area designated by the three factors gives the aggregated distribution area, formally:

$$A(Tmin;P)=1({T}_{min})-\mathop{\sum }\limits_{i=1}^{12}\,0({T}_{min})\,\cap 1(P)-\mathop{\sum }\limits_{i=1}^{12}\,0(P)$$

where *A(Tmin*; *P)* shows the potential distribution area of the given species, which contains the remaining areas after taking into consideration the temperature and precipitation limitations.

**Figure 1:**
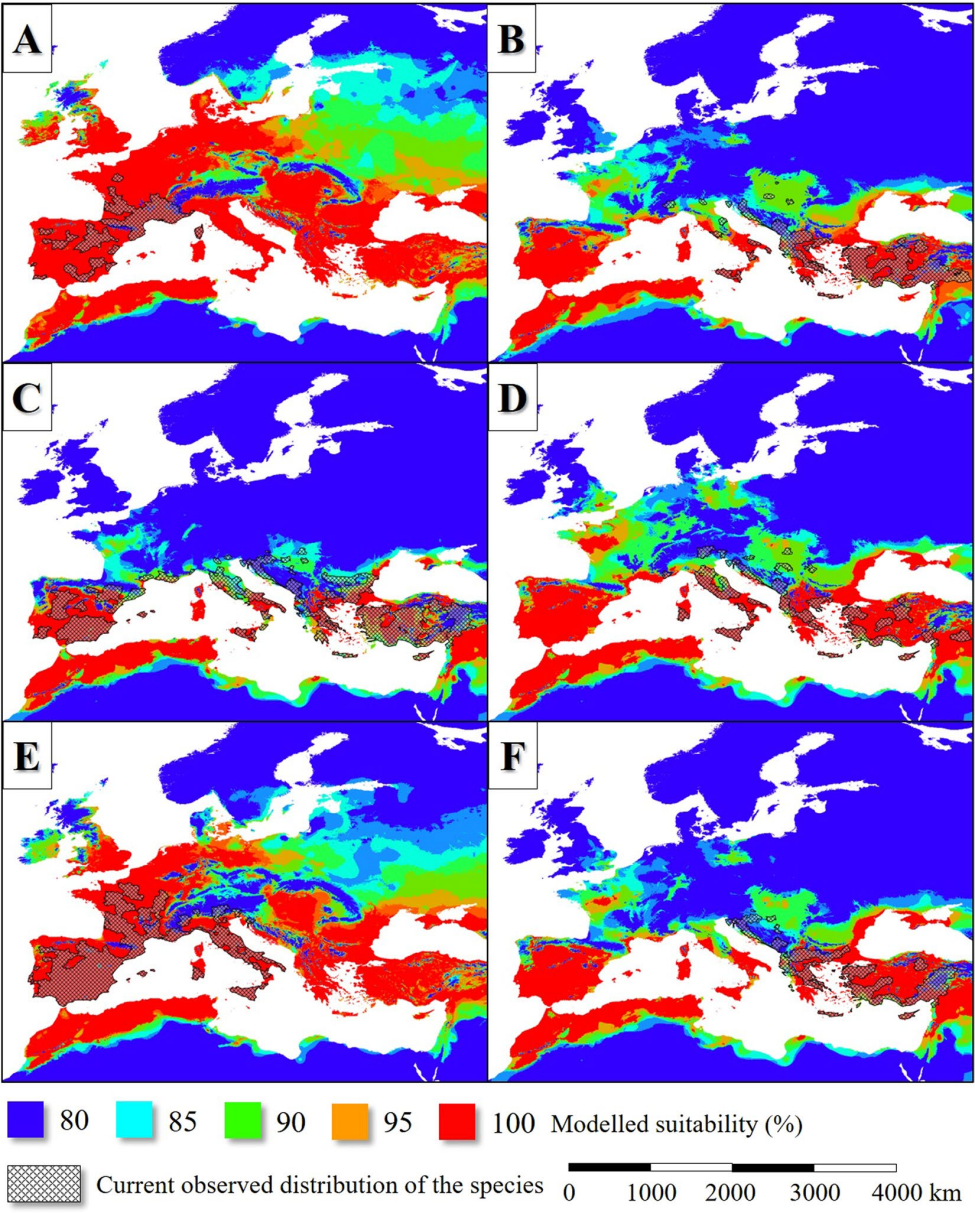
The current observed and the potential distribution of *Ph. ariasi* (**A**), *Ph. neglectus* (**B**), *Ph. papatasi* (**C**), *Ph. perfiliewi* (**D**), *Ph. perniciosis* (**E**) and *Ph. tobbi* (**F**).

In the case of each models (36 per model), the individual presence-absence maps were summarized. The gained values in percentage were held as suitability vales. Comparing the modelled and observed distributions of the species, the 80% suitability value seemed to be the best predictor of the occurrences (Fig. 1). When presenting the results, the colour bar was fit to the suitability range of 80 to 100%.

## Results

### Last interglacial period

**Figure 2:**
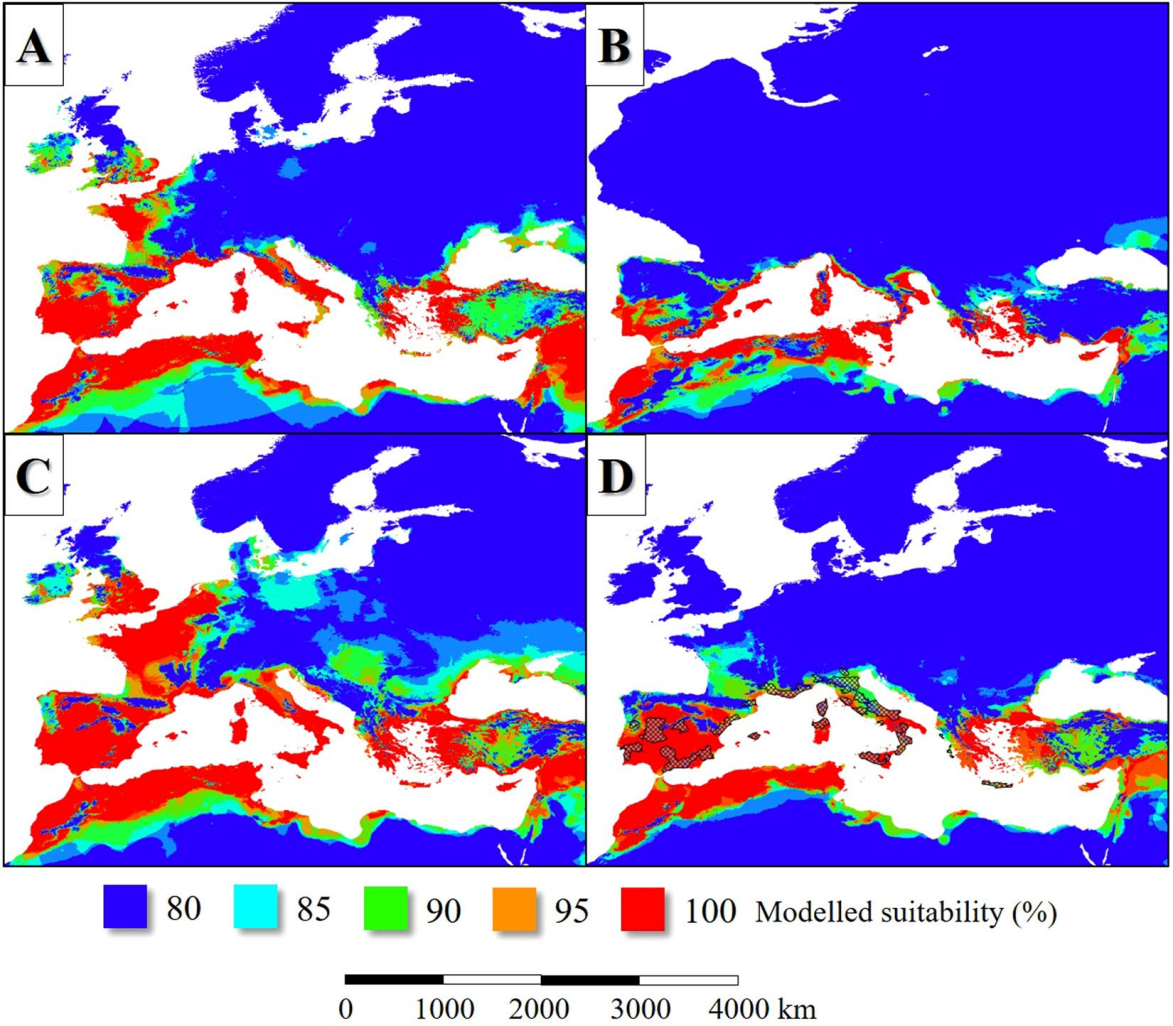
The potential distribution of *L. infantum* according to the climate simulations. (LIP: **A**, LGM: **B**, MHP: **C**, current: **D**) with the current observed range of the parasite in Europe (in **D**).

**Figure 3:**
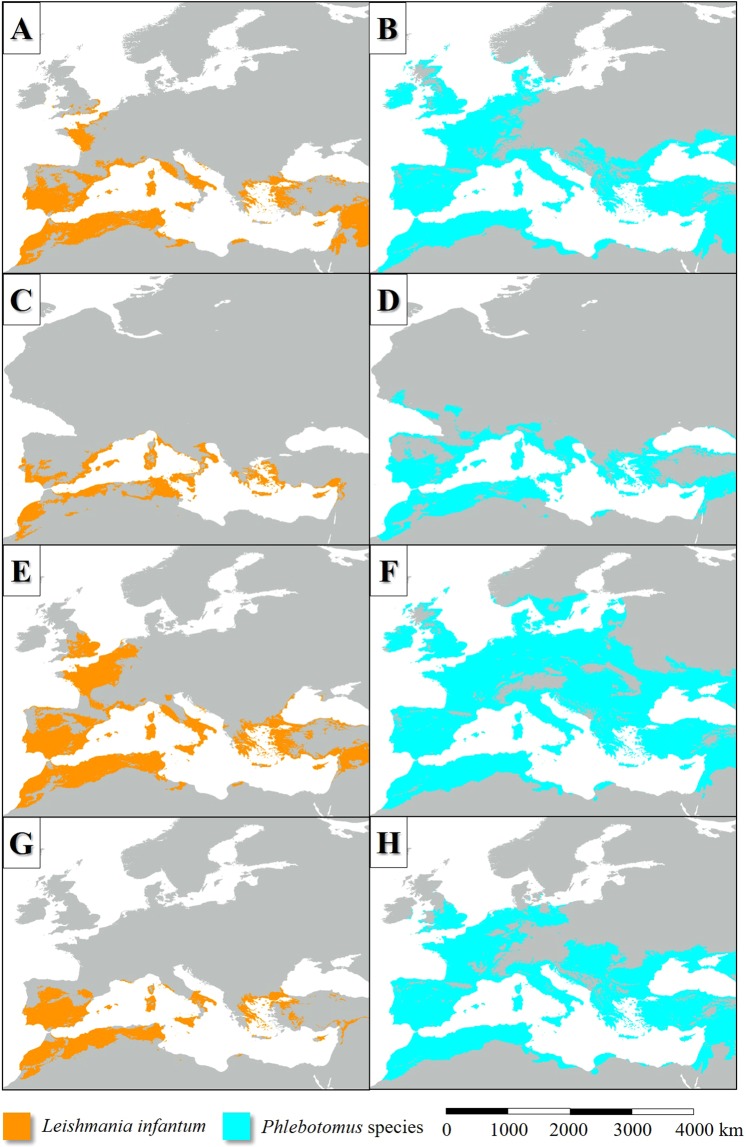
The potential distribution of *L. infantum* (**A**: LIP, **C**: LGM, **E**: MHP, **G**: current) and the summarized potential distribution of the six Mediterranean *L. infantum* vector sandfly species (**B**: LIP, **D**: LGM, **F**: MHP, **H**: current) according to the climate simulations.

**Figure 4:**
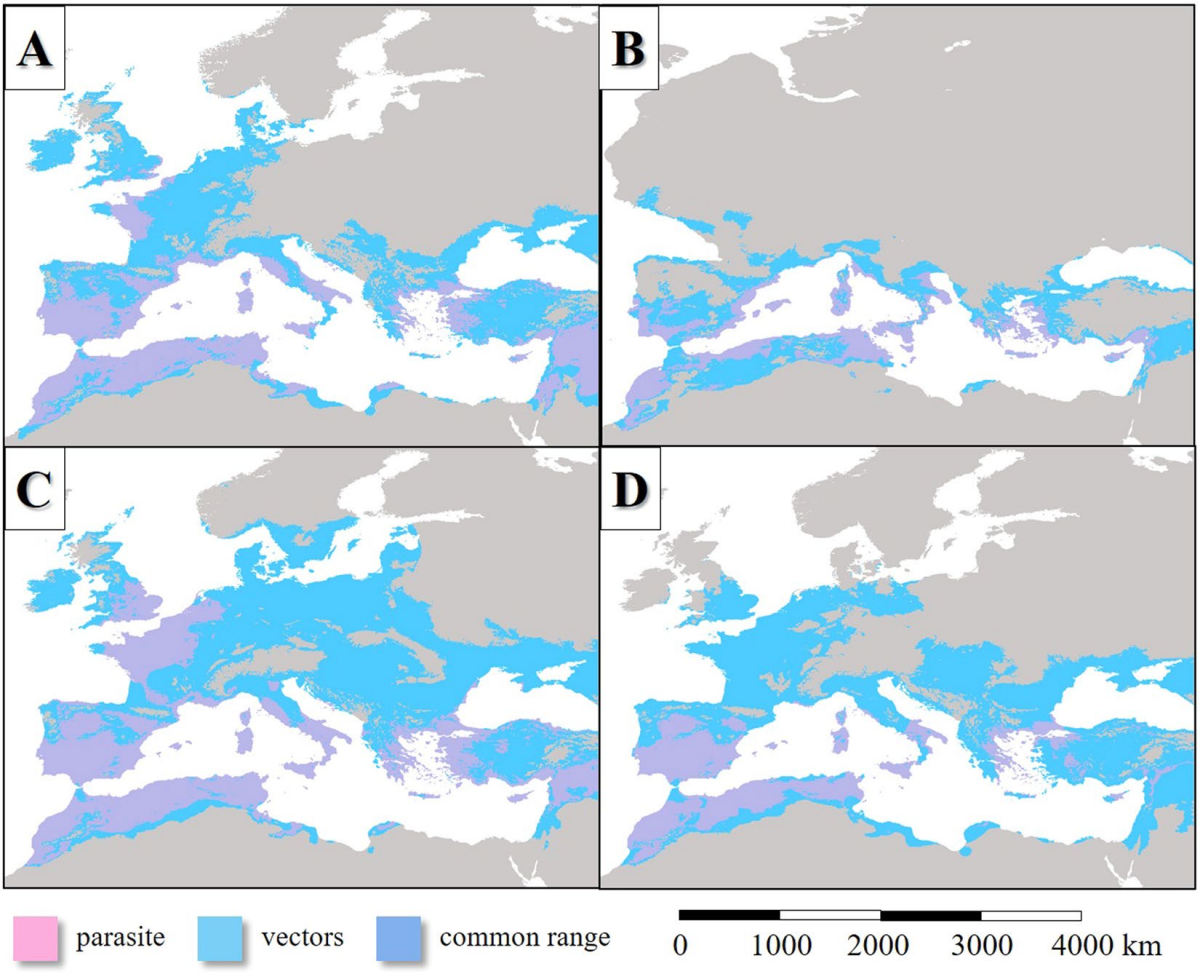
Projection of the potential distribution of *L. infantum* and the summarized potential distribution of the six Mediterranean *L. infantum* vector sandfly species (**A**: LIP, **B**: LGM, **C**: MHP, **D**: current) according to the climate simulations.

In the LIP, the potential distribution of *L. infantum* endemic areas was more extensive than today (Figs 2, 3A), covering mainly the Fertile Crescent, the Atlas Mountains, certain regions of the Apennine Peninsula and Asia Minor, Sardinia and Corsica, Cyprus, Sicily, the lowland areas of the Iberian Peninsula and the coastal regions of the English Channel. Based on the simulation, the parasite was absent from the higher elevations of the Apennine Peninsula and the inland areas of the Balkan Peninsula (Figs 2, 3A). In general, the potential distribution of the parasite was smaller than the range of its vector sandfly species (Fig. 4).

**Figure 5:**
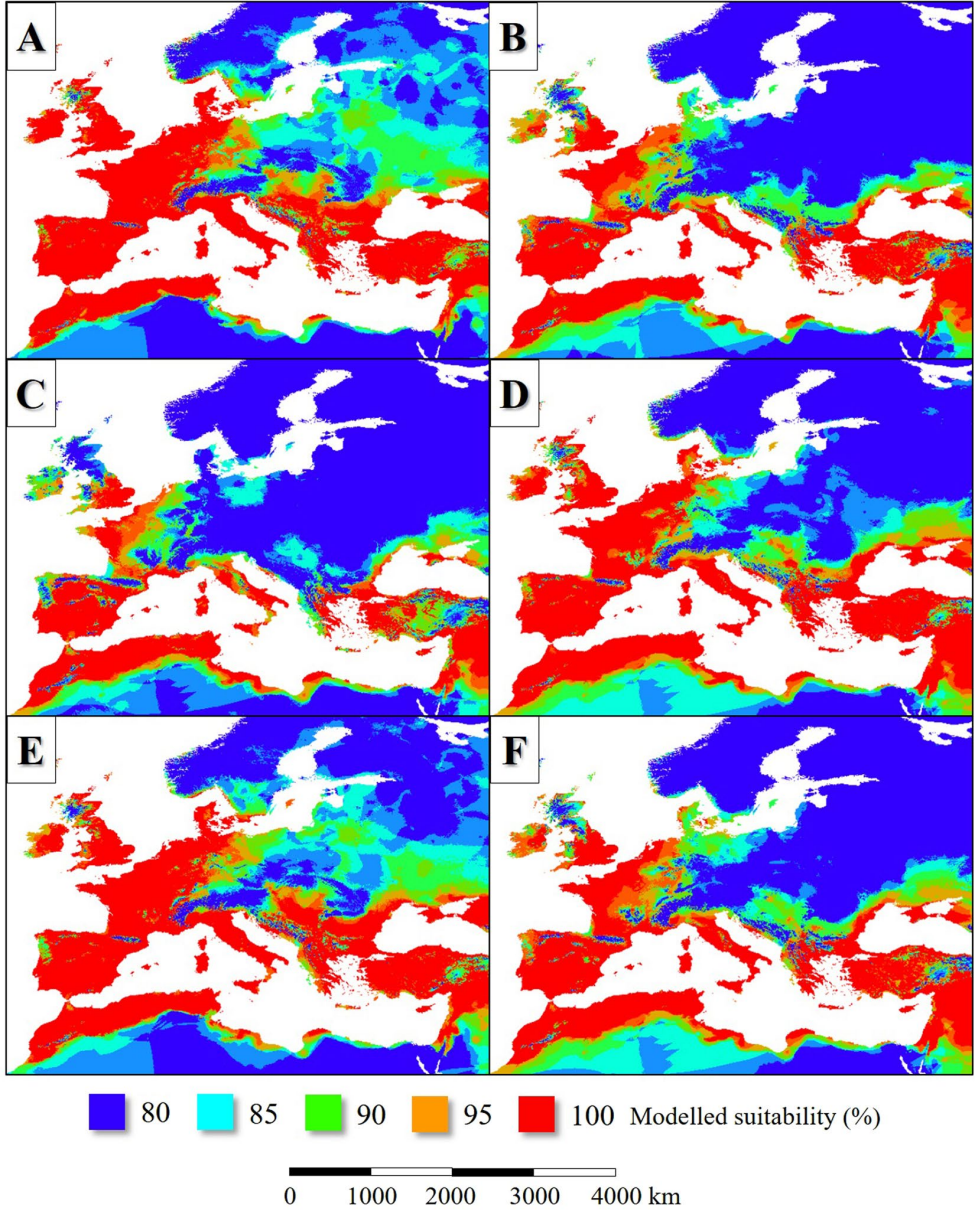
The potential distribution of *Ph. ariasi* (**A**), *Ph. neglectus* (**B**), *Ph. papatasi* (**C**), *Ph. perfiliewi* (**D**), *Ph. perniciosis* (**E**), *Ph. tobbi* (**F**) during the LIP.

According to the model result, about 120–140 ka bp the potential former range of the modelled six *Phlebotomus* species also was slightly smaller-scale than the present-day (Fig. 3B). The Western European distribution was nearly continuous from the coastal region of continental Northwestern Europe to the southwest coasts of the Iberian Peninsula. That time the climate of the British Isles and the northwest part of Europe was suitable for the studied sandfly species (Fig. 5). The climate of the Apennine Peninsula was hostile for sandfly species. The potential Corsican and Sicilian habitats were separated from each other. Northwest Africa understood under this the ranges of the Atlas Mountains formed an extent sandfly distribution area from the west coasts of present-day Morocco to the east coasts of Tunisia. *Phlebotomus* species plausibly neither colonized the northeast coasts of Libya nor Egypt continuously which regions were too arid for sandfly species that time. In the coastal areas of the Levant and the Fertile Crescent, wide areas were suitable for *Phlebotomus* species. Most of the areas of Asia Minor, the coastal plains and islands of the Aegean Sea were potentially sandfly-inhabited areas. Potential *Phlebotomus* habitats were in the coasts of the Black Sea including the coasts of Sea of Azov and the Crimean Peninsula (Fig. 3B).

**Figure 6:**
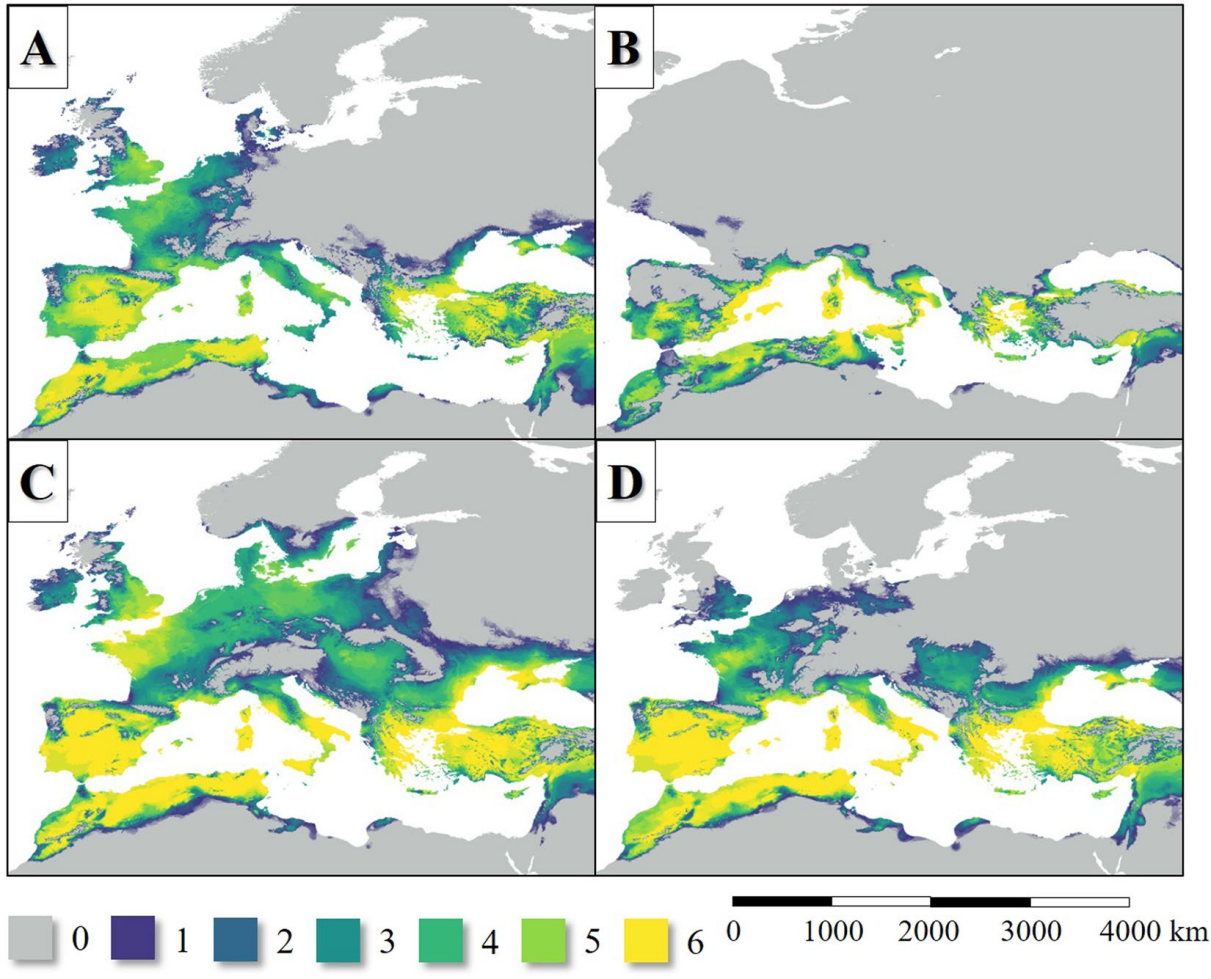
The potential diversity of the six Mediterranean *L. infantum* vector sandfly species during the LIP (**A**), LGM (**B**), MHP (**C**) and the current period (**D**).

The potential diversity of the *L. infantum* vector sandfly fauna was the highest in the Iberian Peninsula, Northwest Africa, the Balkan Peninsula and Asia Minor area with about 5–6 species, in general. In the Apennine Peninsula, the average potential species number was 2–4 species, while in the border regions it could be no more than 2 species during the LIP. The presence of sandfly species in the British Isles is questionable (Fig. 6A).

### Last glacial maximum

Based on the simulation, the distribution of *L. infantum* was restricted to the coastal areas of the Mediterranean Sea and it was absent from the Fertile Crescent (Fig. 3D). While the past distribution of the parasite was smaller than its vectors (Fig. 4B)’, the potential area of *L. infantum* during the LGM was restricted mainly to the continental shelf of the Mediterranean landmasses (Fig. 2B). Based on the CCSM4 climate simulation, during the LGM 21 ka bp, two major, notable sandfly refugia existed in the West and East Mediterranean Basins and did not formed continuous distribution area (Fig. 3C). The coastal areas of the Libyan desert, the Shinai Peninsula were not inhabited by the parasite due to the glacial aridity of the Sahara.

**Figure 7:**
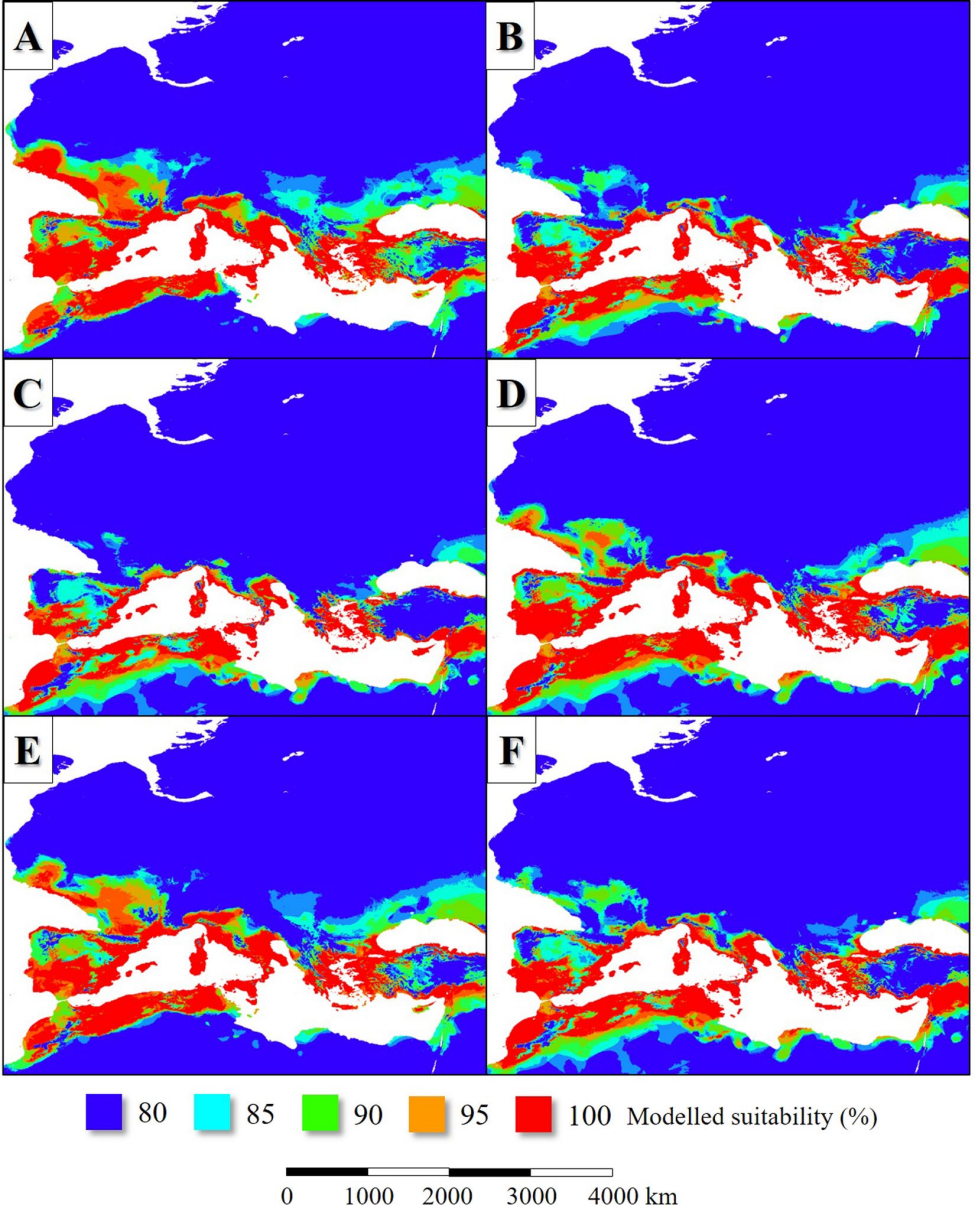
The potential distribution of *Ph. ariasi* (**A**), *Ph. neglectus* (**B**), *Ph. papatasi* (**C**), *Ph. perfiliewi* (**D**), *Ph. perniciosis* (**E**), *Ph. tobbi* (**F**) during the LGM.

In the case of the *Phlebotomus* species, the suitability values also were the highest in the Mediterranean coasts (Fig. 7). The Aegean area formed various refugium in the geographical sense. The coastal areas of Southeast Balkan, the now sunken landmasses of the Aegean Sea and the western parts of Asia Minor with the southwest coastal areas of the Black Sea potentially could be inhabited by sandfly species. The Middle East refugium covered the southern coastal areas of Asia Minor, Cyprus, the northernmost parts of the Levant and the other western areas of the Fertile Crescent (Fig. 3D). The north part of the West Mediterranean refugium formed continuous distribution area including the European coastal areas of the Alboran, the Balearic, the Ligurian, the Tyrrhenian, the Sicilian, the Ionian, and the Adriatic Seas. The cool climate of the South Dinarids dissected the West and East Mediterranean sandfly fauna.

Due to the low glacial sea level, land bridges and narrow sea straits existed in the LGM. The former Gibraltar strait was narrower than the present-day. Almost continuous migration path existed between the Southeast Balkan Peninsula and Asia Minor through the former Aegean landmasses (inc. the landmass of the Cyclades) with narrow straits. The Ionian strait between the continent and Crete also was narrower than today. A land bridge existed between Corsica and Sicily and very narrow strait or land bridge between Corsica and the Apennine Peninsula. A very narrow strait existed between Sicily and the Calabrian Peninsula. The Sicilian strait was much narrower than today with several minor islands which created a continuous migration pathway for such fledged creatures such are sandfly species (Fig. 3D).

In the LGM, the potential *L. infantum* vector sandfly diversity of Sicily was higher (5–6 species) than in the case of the LIP, due to the dryer climate of the Mediterranean (compare Fig. 6A,B). Similar high modelled diversity can be seen in the case of the South Iberian and the Aegean area. In the Iberian Peninsula and the Atlas Ranges, the average number of *L. infantum* vector *Phlebotomus* species could be lower compared to the LIP’s stage due to the notable cooling of the higher elevations (Fig. 6B).

### Middle holocene period

Based on the model simulation, *L. infantum* was present in many areas of the Mediterranean Basin during the MHP, although the Iberian and the northwest distribution area were the most extensive that time. Although the potential distribution of the parasite during the MHP seems to be much smaller than the distribution of its potential vectors (Fig. 4C), the difference is somewhat smaller than in the case of the present era (Fig. 4D). It is due to the modelled potential presence of the parasite in Western Europe.

**Figure 8:**
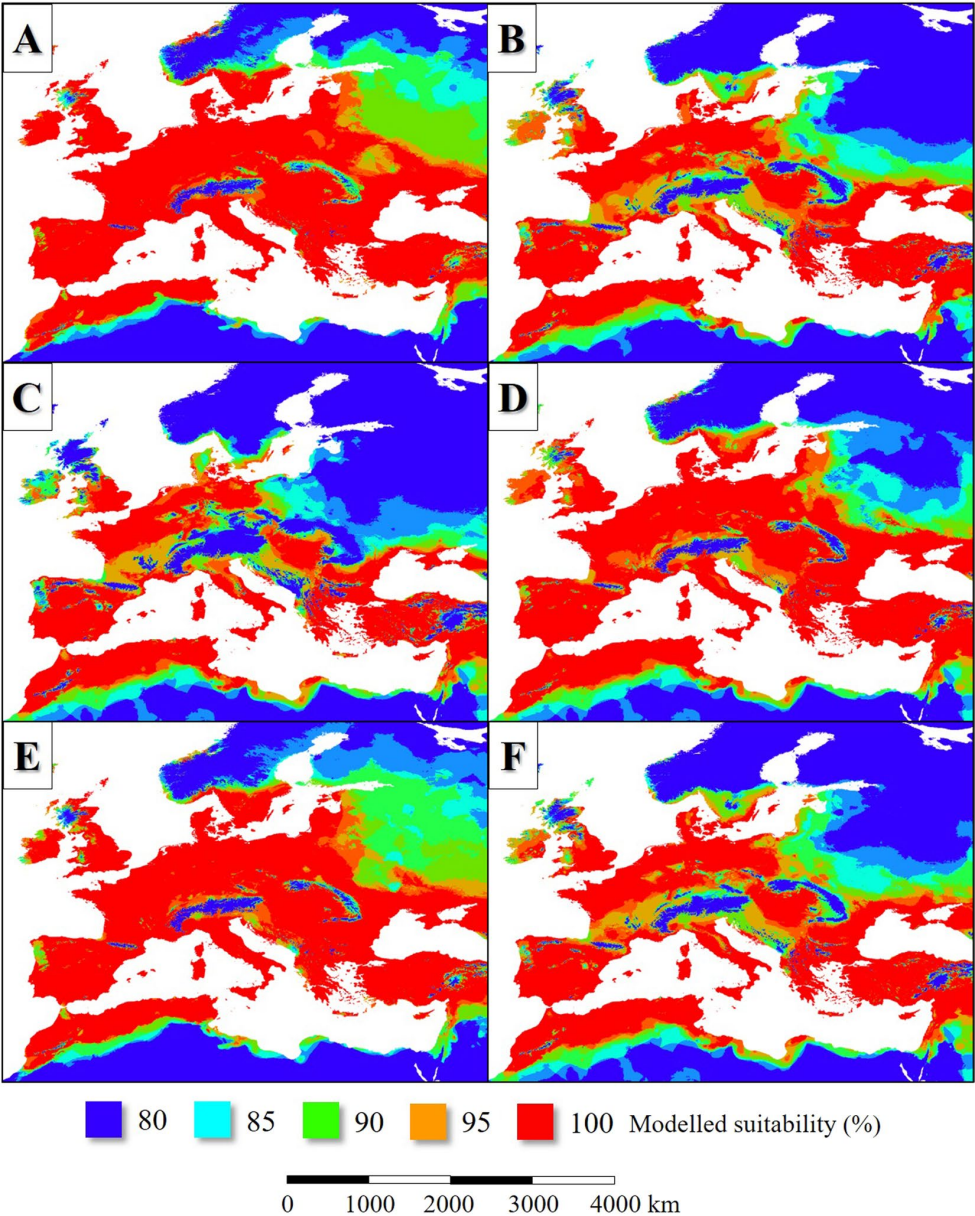
The potential distribution of *Ph. ariasi* (**A**), *Ph. neglectus* (**B**), *Ph. papatasi* (**C**), *Ph. perfiliewi* (**D**), *Ph. perniciosis* (**E**), *Ph. tobbi* (**F**) during the MHP.

In the MHP, the potential distribution area of the sandfly vectors was much extent than in the present days in the case of all species (Fig. 8). Extent potential sandfly habitats existed in West and Central Europe and even in the southern parts of Scandinavia. The summarized potential sandfly distribution range (Fig. 3F) was much more extent than today including such areas like the present-day Benelux states, North Germany, the east coasts of Denmark, North Poland and the east coasts of the British Isles. Also, large potential sandfly distribution area existed around the Black Sea. The habitats of the Apennine Peninsula were in direct contact with the Iberian habitats. In the case of North Africa, the Aegean area, Asia Minor and the Fertile Crescent, the potential distributions during the MHP were similar to the situation of the LIP (compare Fig. 3B,F). The model also shows continuous sandfly habitat area in the Carpathian Basin, and the adjacent Moravian Plain and some other parts of eastern Europe.

In the MHP, the climate of large areas of West and Central Europe were suitable for the vectors of *L. infantum*, although the diversity was lower (2–5 species) than in the Mediterranean Area. As in the earlier periods, it was shown, the diversity of the East Mediterranean Basin and the surrounding landmasses could be the highest. The model shows that *Ph. ariasi, P. perfiliewi* and *Ph. perniciosus* potentially could inhabit such northern areas as the Danish Peninsula. Diverse sandfly fauna (5–6 species) could exist in the south regions of the Apennine Peninsula and connection may have established between the sandfly populations of the Fertile Crescent and the Aegean region. The presence of West Mediterranean sandfly species in the Carpathian Basin is plausible that time, however, the connection between the West and East Mediterranean area (Fig. 3C).

### Area changes in the last 140 kys

**Figure 9:**
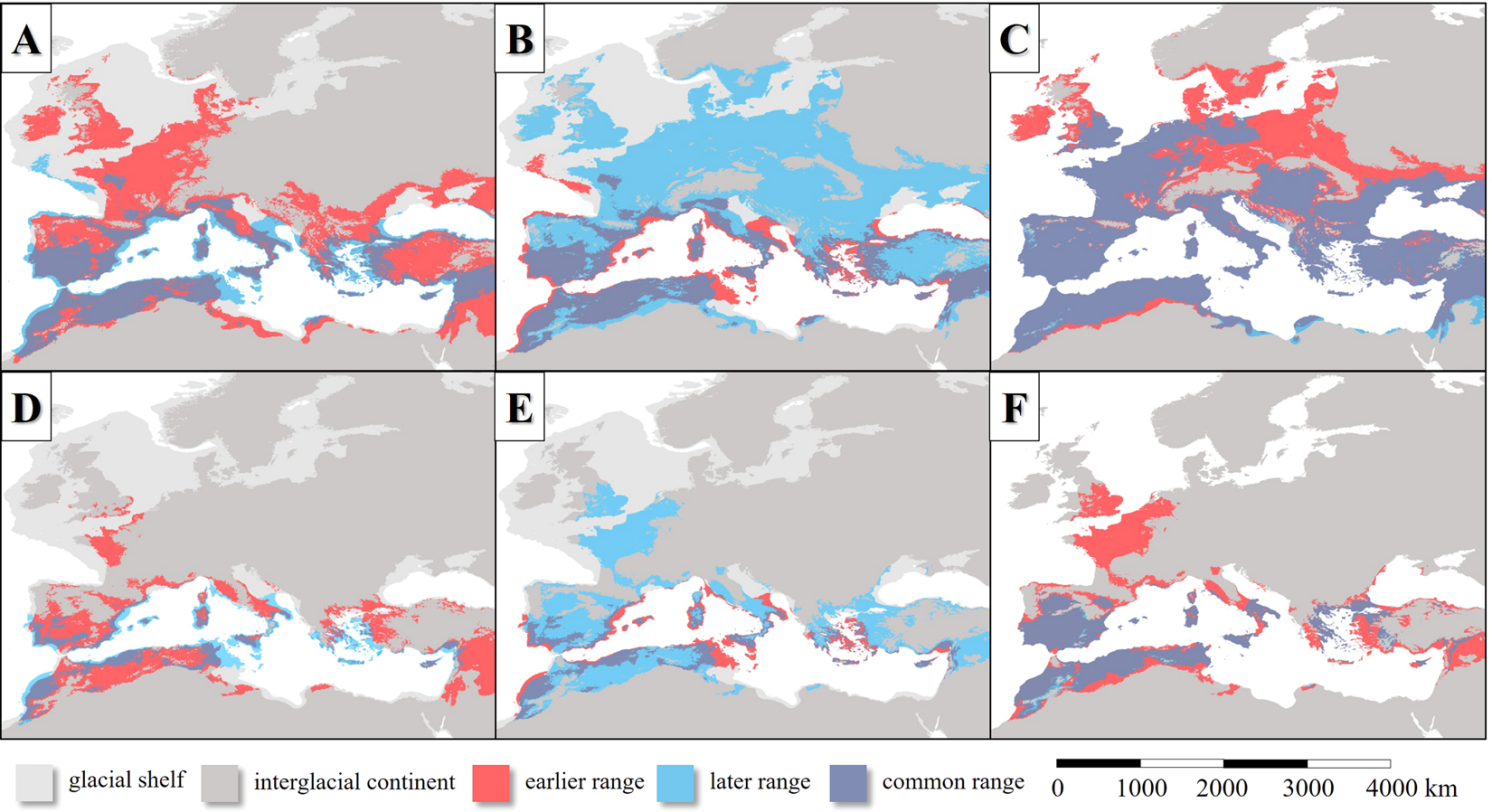
Projection of potential distribution of the six Mediterranean *L. infantum* vector sandfly species (**A**–**C**) and *L. infantum* (**D**–**F**) in the comparison in the following time intervals: the LIP to the LGM’s and the LGM to the MHP’s and the MHP to the current’s potential distribution patterns.

The change of the potential distribution areas showed different patterns in the north and the south coasts of the Mediterranean Basin in the case of *Phlebotomus* species. In the southern coastal areas of the Mediterranean Sea, a moderate range contraction of habitats can be seen between the LIP and the LGM (Fig. 9A). In the shelves of the West Mediterranean Basin, the areal gain was more notable than the range loss due to the cooling climate. In contrast, to the Mediterranean coasts, the last glacial cooling vanished the sandfly species from large areas in the northern distribution area and caused the southward shift of the species from their former extent habitats to the narrow margins of the shelf of the Mediterranean Sea. Similar phenomena could happen in the Balkan and Asia Minor. Except for the Iberian Peninsula, the Atlas Mountains and the Fertile Crescent, the inland habitats disappeared due to the cooling. After the LGM, the varying in time, but continuous sea level rise caused the notable contraction of the sandfly habitats in the Aegean Sea and around the Strait of Sicily and the Adriatic habitat area also suffered hundreds of kilometers of retreat (Fig. 9B). Between the LGM and the MHP, the habitat gain was notable in West Europe and the South Baltic area, in the Carpathian Basin and around the Aegean and the Pontic area. After the MHP, the slow cooling trend of the last 6 kys resulted in the retreat of the *L. infantum* vector sandfly taxa from the north and northwest regions of Central and West Europe (Fig. 9C). Un the Mediterranean area, where the climate remained still warm and dry enough for sandfly species, the potential distribution range did not change. The changes of the potential distribution patterns of the parasite show the parallel changes to its vectors. Both in the LIP and MHP the potential range of *L. infantum* covered certain parts of Western Europe. It is common in the case of LGM and the current stage, that these Western European potential range is absent in the modelled maps. Because, both the past and the current distributions of the parasite are much less extent compared to the vectors. It can be concluded, that the level of the absolute habitat changes was not so significant as in the case of the vectors (Fig. 9D–F).

## Discussion

At first, it is very important to be discussed whether the recent niche of the studied sandfly species was similar to their Pleistocene ancestors or not. It is the basis of the evolution theory that species either adapt to the changing environmental circumstances or migrate to the more suitable areas or die out. The rapidly changing climatic conditions of the Pleistocene era caused the radical and repetitive shifts of the biome borders in South Europe and North Africa^57^. For example, during the LGM, broadleaved forest was confined to small refuges on the eastern coast of the Black Sea^58^ which region today has humid subtropical, *Cfa* and oceanic *Cfb* climate according to the Köppen-Geiger climate classification system^59,60^. In the present days, this area belongs to the range of *Ph. papatasi*^61^ which species may survive the extremely cold periods of the LGM in the narrow, southern coastal regions of the Black Sea according to our model results. Many speculations about the past adaptive migrations and speciation of the sandfly ancestors were directly or indirectly based on the hypothesis that the recent climatic requirements of the species can be rather the fingerprint of the climatic circumstances of the long Tertiary period that the result of the relatively short Pleistocene era^44,45,62^. These studies hypothesized or found the relative niche conservativism of sandfly taxa during the Neogene and Quaternary era. Other studies showed that sandfly species retreated to their Iberian, Apennine and Balkan refugees during the coldest periods^3^, and their repeated dispersal occurred in the start of the interglacial as it could happen in the case of the youngest postglacial warming period^34,41^. These facts rather reflect that sandfly species always inhabited the warm temperate climate habitats in Europe and followed the changes of the biome borders. In the side of the parasite, if we accept the hypothesis of Noyes (1998) about the neotropical origin of the genus *Leishmania*^63^, it is plausible that *Leishmania* species inherited their cold-sensitivity from their tropical ancestors. It could explain the present cold-sensitivity and the equatorial-tropical-subtropical occurrence of these parasites.

Our findings confirm the hypothesis that such West Mediterranean species as *Ph. ariasi* could have populations north of the Iberian Peninsula^3^. Comparing the variability of the Moroccan, Iberian and South France populations of *Ph. ariasi*, the presence of a refugium north of the Pyrenes during the LGM was suggested^3^. The recent populations of *Ph. ariasi* in the Mastiff Central could be the direct descendants of the coastal *Ph. ariasi* populations in the Gulf of Lion. Comparing the modelled potential habitats of the West Mediterranean species in the LGM and the MHP it can be concluded that the genetic evidence suggested sandfly habitats existed in the Gulf of Lion and could form the origin of the Holocene expansion of the West Mediterranean taxa. Since the two West Mediterranean character species *Ph. ariasi* and *Ph. perniciosus* can be found both in Southwest Europe and North Africa, it is inevitable that these species could pass the ocean through Gibraltar or the Sicilian Strait.

In the recent times, Europe and Africa are separated by 14.3 km sea at the Gibraltar strait between the Rif Cordillera Baetics and the narrowest point, which naturally was also narrower than the present during the LGM. In the case of *Ph. perniciosus*, two genetically distinct lineages exist: the ‘typical’ in Morocco, Tunisia, Malta and Italy, the Iberian in Southern Spain. The two regional groups of *Ph. perniciosus* populations remained isolated in the western Mediterranean subregion, while recently within each region there are no significant permanent barriers to gene flow between contiguous populations^34^. During the Pleistocene Ice Ages, *Ph. perniciosus* could have survived in western Europe, but continuously in refugia only in Southern Spain and Southern Italy and it is died out in France and in the northern areas where temperatures were too low for adult activities in the summer^41^. Our model results do not provide direct evidence for the existence of two distinct sandfly refugia in Iberia and the Apennine Peninsula, but the narrow coastal foothills of the Maritime Alps and the Mediterranean coasts of the Pyrenes could form a relative barrier.

The extent and continuous West Mediterranean refugium can explain the fact why the West Mediterranean populations of the West Mediterranean *Ph. perniciosus* are relatively homogenous in a genetic and morphologic sense. The populations of these species sampled from Tunisia, Malta, and Italy also showed low haplotype diversity^64^. We can only partially agree with the hypothesis^42^ that the Mediterranean Sea is not an effective barrier to prevent the dispersal of *Phlebotomus* species. Each of the modelled distributions shows that during the colder periods there was no communication between the sandfly faunae of the Apennine and the Balkan Peninsulas. However, the recent relative separation of the West and East Mediterranean may be much older. It plausible, that the wide but disjunctive recent range of the Trans-Mediterranean species *Ph. papatasi* can be the remnant of the former, continuous distribution, plausibly in the pre-Pleistocene times^43^, because the first glacial and interglacial periods were milder in the first part of the Quaternary than the later ones in the last 500 kys^65^.

Surprisingly, it was found that Asia Minor, which is the habitat of several sandfly species in the recent times, was highly sensitive to glacial cooling, despite the fact that this peninsula has a similar latitudinal position like the Iberian. The climatic stability of the Iberian Peninsula is the consequence of the Gulf stream which climatic influence is weak in the Eastern Mediterranean Basin. Therefore, only certain coastal areas of the Asia Minor Peninsula were suitable for sandfly species in the LGM. Mountain ranges of a considerable altitude could form absolute barriers between sandfly populations in the colder phases of the Pleistocene, isolating the sandfly populations of South-eastern Anatolia^66^. Because our model indicates the moderate sandfly habitat retreat of the Middle East and even more the notable reduction of the Asia Minor refugium during the LGM, the observed differences in the haplotype diversity can be the consequence of the glaciation which is rhymed with the fact that the main factor determining the degree of genetic diversity of the Trans-Mediterranean *Ph. papatasi* was latitude and not climatic conditions^67^. However, it can be accepted that the Mediterranean ranges formed significant barriers between the sandfly populations of the adjacent refugia in the LGM, the connection between the latitude and climate is obvious and the distinction between the two main abiotic factors may seem somewhat artificial.

There are less or more uncertainties in the reconstruction of the former diversity patterns. In general, the presence of the West Mediterranean *Phlebotomus* species in the British Isles and Scandinavia is questionable because nowadays channels separate these landmasses from the continent. The used climate models contain a kind of topographical ‘reconstruction’ of the former shorelines. While the LGM shorelines were based on the topography of the shelves under glacial sea level conditions, the shortcoming of these maps that LIP and MHP shorelines simply follow the recent topographical patterns. Before the Pleistocene Era and during the glacial maximums, the British Isles were part of continental Europe. This usually does not cause a big mistake because the post-glacial sea level rise almost reached its plateau phase 7,000 yrs BP and the sea level rise was about only about 2 meters in the last 6000 ys^68^. On the other hand, in the last 10 kys, the global sea level has never been higher than it is at present^69^. In the other hand, during the LIP, the sea level was very similar to today. The difference was only a few meters^70^.

While it can be stated that the sea level differences do not invalidate the models, other factors can strongly influence its accuracy: the so-called Weald-Artois Anticline Ridge created a direct connection between the Isles and continental Europe. It is known that the ends of the glacial periods, the ridges acted as a natural barrier, blocking the runoff the meltwater of the Dogger Bank region. The ridge was gradually broken by the subsequent episodes of catastrophic glacial lake outburst floods^71,72^. It is also known that the shoreline of the Littorina Sea 7,500–4,000 BP were different from the shoreline of the derived Baltic Sea since this ancient sea covered more surface area than the today’s Baltic Sea. In the other hand, not only the post-glacial uplift changed the topography, because the sea rearranged the material of the glacial moraines, broken down and rebuilt them in other places creating spits and lagoons. These circumstances cause notable uncertainties in the reconstruction of the shoreline of the North Atlantic and Baltic area in the MHP^73^. It can be concluded that the English Channel and the Danish straits did not exist in its recent form in the LIP and the MHP and it is possible that there was a direct connection between the continent and the opposite coasts episodically.

To discuss the potential bias of the study is very important. Although the model prognoses habitats for Western European sandfly species in Eastern Europe in the past, the mechanistic acceptance of this model result somewhat seems to be misleading. Unfortunately, neither can we prove, nor can refute whether a species in the past did not actually occur in an area. The prediction extra-areal distributions are not a problem in the case of future predictions because the anthropogenic transport can to overcome geographical barriers nowadays. In contrast, in the past, the anthropogenic sea, air and land transport or tunnels under the mountain ranges did not exist. In a zoogeographical sense it would be substantially false to project the past distribution of species on those regions, where recently they cannot be found. In the case of sandfly species, we are in the position that several authors investigated the past plausible distribution of these species and concluded that mountains and seas formed notable barriers against sandfly migration. For example, such mountain ranges as the Pyrenes which often formed barriers for dispersals^3,34^ during the warming postglacial phases. Based on this fact - accepting the clear uncertainties of the approach, the total potential diversity of the L. infantum vector sandfly taxa were depicted in the figures even we know, that the past diversity values could have been lower, especially in the Western Mediterranean area.

As it was discussed, genetic studies provided indirect evidences for the former existence of different sandfly species in the Mediterranean area. It can be hypothesized that if the recent range of a species covers a known refugium, the species plausibly occurred in this habitat also during the LGM. As the model results showed, the milder climate of Holocene climate optimum may result in the surprisingly great expansion of the West Mediterranean species in West and Central Europe. *Phlebotomus* species have been found in several parts in Belgium and Germany which observations originally were thought to be the consequence of the recent global warming^2^. The West Mediterranean populations of *Ph. perniciosus* persisted since the Holocene climate optima in Central Europe sporadically. It should be also noted that the shorelines significantly differed from the recent one in the LGM. About 22 kys BP. during the LGM, sea level was about 125 meters (about 410 feet) lower, while the average global temperatures were about 5.5 colder than it is today^74^. Most of the British Isles, Scandinavia, and the Baltic coasts were covered by the Fennoscandian Ice Sheet. Large areas of the present-day West, Central and East Europe were covered by periglacial polar desert. From the south, the so-called mammoth steppe extended from the Iberian Peninsula to the Far East.

The potential anthropologic consequences of this study cannot be neglected. Based on the modelled distributions, archaic human populations of Europe could be less influenced by the sandfly-transmitted diseases than the human populations of North Africa and the Middle East. In the late second part of the Middle Palaeolithic Period, the Neanderthal populations of the Apennine Peninsula^75^ could be influenced by sandfly-borne diseases even not by *L. infantum* parasite. In contrast, the Northwest Balkan population of Neanderthals^76^ lived in a sandfly-free area. Since both in the LIP and the LGM the Fertile Crescent could be the home of *L. infantum* and its vectors, it has been probable that the archaic *Homo sapiens* sapiens^76^ and the Neanderthal populations^77^ in the Middle East lived in a partly sandfly-inhabited area. In the Upper Palaeolithic Period, the last Neanderthal populations of the Iberian Peninsula^76,78^ theoretically could be infected by sandfly-borne diseases because sandfly populations have relatively extent potential area both in the LIP and the LGM.

## Conclusions

Leishmaniasis, which is transmitted by hematophagous females of *Phlebotomus* and *Lutzomyia* species^79^ claiming more than 50,000 lives annually^80^ and about 0.7 million to 1.3 million new cases occur worldwide annually^81^. For this reason, it was found to be an important task to gain more information for the dispersal capacity and the historical causes of the recent phylogenetic and distribution factors of the vector sandfly species and *L. infantum* in the Mediterranean Basin and the continental regions of Europe. It was shown that in the last 140 thousand years, the potential sandfly habitats and the occurrence of *L. infantum* around the Mediterranean Sea suffered notable alterations. Although the Balkan Peninsula was more sensitive to climatic fluctuations than the Iberian refugium, it is plausible that the outlined coastline and the many islands of the Aegean Sea provided more stable conditions for survival during the coldest episodes. The modelled glacial sandfly refugees are strongly supported by the former phylogenetic evidence.

## Data Availability

The datasets generated during and analysed during the current study are available from the corresponding author on a reasonable request.

## Supplementary information

Supplementary table 1.
