# Supplementary material for: The changing distribution of Leishmania infantum Nicolle, 1908 and its Mediterranean sandfly vectors in the last 140 kys
Source: Sci Rep. 2019 Aug 14;9:11820. doi: 10.1038/s41598-019-48350-7 (PMC6694126; doi:10.1038/s41598-019-48350-7)
Supplement: Supplementary file 1 — Supplementary table 1. [file 41598_2019_48350_MOESM1_ESM.pdf]

The changing distribution of *Leishmania infantum* Nicolle, 1908 and its Mediterranean sandfly vectors in the last 140 kys

Attila J. Trájer[1*], Viktor Sebestyén[2]

[1]University of Pannonia, Department of Limnology, H-8200, Veszprém, Egyetem utca 10.

[2]University of Pannonia, Institute of Environmental Engineering, H-8200, Veszprém, Egyetem utca 10.

*Corresponding author's e-mail address: trajer.attila@mk.uni-pannon.hu, tel./fax. (+36)88-62-6116; ORCID: 0000-0003-3248-6474

Supplementary materials

Supplementary table 1. The climatic factors of the modelled species.

Species	Factor	Months											
		1	2	3	4	5	6	7	8	9	10	11	12
Ph. ariasi	Tmin, °C	-0.6	0.3	2.4	5.3	9	12.9	15.1	14.7	12.4	8.1	3.4	1.2
	Pmin, mm	21	21	27	21	15	6	0	3	15	27	30	21
	Pmax, mm	183	141	159	156	138	96	87	84	93	150	186	156
Ph. neglectus	Tmin, °C	-2.4	-1.7	2.7	6.8	10.8	15.3	18.5	18.2	14.6	9	4.3	-1.5
	Pmin, mm	24	24	21	15	6	0	0	0	3	9	15	24
	Pmax, mm	207	144	138	120	78	54	45	45	72	114	189	171
Ph. papatasi	Tmin, °C	-0.5	0.3	3.8	7.5	11.6	16	18.7	18.8	15.3	9.9	5.3	0.3
	Pmin, mm	18	18	15	12	6	0	0	0	0	6	12	18
	Pmax, mm	177	120	129	114	81	57	45	42	66	108	162	138
Ph. perfiliewii	Tmin, °C	-2.3	-1.3	2	5.8	10	14.5	17.4	16.9	13.6	8.4	3.7	-0.9
	Pmin, mm	18	21	15	12	6	0	0	0	0	6	12	18
	Pmax, mm	216	147	144	132	90	66	51	57	90	141	216	171
Ph. perniciosus	Tmin, °C	0.5	1.4	3.4	6.2	9.8	14.1	16.1	15.9	13.6	8.8	4.2	2.1
	Pmin, mm	18	18	24	18	12	3	0	3	9	18	24	21
	Pmax, mm	171	117	144	135	117	84	78	81	81	129	171	126
Ph. tobbi	Tmin, °C	-2.7	-1.8	2.5	6.5	10.6	15.1	18.3	18.1	14.3	8.9	4.2	-1.9
	Pmin, mm	18	21	15	12	6	0	0	0	0	6	12	18
	Pmax, mm	216	150	141	120	78	66	51	51	69	114	192	174
L. infantum	Tmin, °C	1.9	2.7	4.8	7.5	11.8	16.5	19.4	19.6	16.1	11	6.1	3.3
	Pmin, mm	18	18	21	15	6	0	0	0	3	9	18	21
	Pmax, mm	183	111	147	120	84	48	39	36	72	120	171	123
